# Population policies, programmes and the environment

**DOI:** 10.1098/rstb.2009.0162

**Published:** 2009-10-27

**Authors:** J. Joseph Speidel, Deborah C. Weiss, Sally A. Ethelston, Sarah M. Gilbert

**Affiliations:** 1 Bixby Center for Global Reproductive Health, University of California, San Francisco, 3333 California Street, Suite 335, PO Box 0744, San Francisco, CA 94143-0744, USA; 2 Population Council, One Dag Hammarskjold Plaza, New York, NY 10017, USA; 3 PATH Malaria Vaccine Initiative, 7500 Old Georgetown Road, Suite 1200, Bethesda, MD 20814, USA

**Keywords:** population, environment, family planning, natural resources, reproductive health

## Abstract

Human consumption is depleting the Earth's natural resources and impairing the capacity of life-supporting ecosystems. Humans have changed ecosystems more rapidly and extensively over the past 50 years than during any other period, primarily to meet increasing demands for food, fresh water, timber, fibre and fuel. Such consumption, together with world population increasing from 2.6 billion in 1950 to 6.8 billion in 2009, are major contributors to environmental damage. Strengthening family-planning services is crucial to slowing population growth, now 78 million annually, and limiting population size to 9.2 billion by 2050. Otherwise, birth rates could remain unchanged, and world population would grow to 11 billion. Of particular concern are the 80 million annual pregnancies (38% of all pregnancies) that are unintended. More than 200 million women in developing countries prefer to delay their pregnancy, or stop bearing children altogether, but rely on traditional, less-effective methods of contraception or use no method because they lack access or face other barriers to using contraception. Family-planning programmes have a successful track record of reducing unintended pregnancies, thereby slowing population growth. An estimated $15 billion per year is needed for family-planning programmes in developing countries and donors should provide at least $5 billion of the total, however, current donor assistance is less than a quarter of this funding target.

## 1. Overview

Population gained acceptance as an environmental issue in the late 1960s and early 1970s, following the publication of Paul Erlich's book *The Population Bomb* (Ehrlich 1968) and the celebration of the first ‘Earth Day’. Yet, more than 30 years later, population seems to have largely dropped off the environmental movement's agenda, owing at least in part to three factors: (i) uncertainty and controversy around population and reproductive health and rights issues, such as those relating to family planning, abortion and various governmental population policies; (ii) the political dominance of a largely anti-environmental White House and Congress in the USA; and (iii) a shifting of priorities within the US environmental movement in response to immediate threats such as loss of biodiversity and climate change.

Parallel to these developments, activists and foreign-aid donors concerned about population policies and programmes increasingly have focused their attention on reproductive health, especially HIV/AIDS, and on ensuring that family planning and other reproductive health programmes respond to the individual needs of women and men and are fully voluntary (Germain & Kyte 1995). Less attention has been focused on the demographic rationale or consequences of population programmes and, consequently, their environmental implications. Like their environmentalist colleagues, family planning and reproductive health advocates in the USA face determined opposition from social and other ideological conservatives, who try to minimize the significance of continued population growth or to limit the medical options of those seeking to avoid pregnancy and sexually transmitted infections (STIs).

Although preservation of natural systems through reduction of the degradation and consumption of natural resources is urgently needed, this paper seeks to refocus attention on the importance of population trends to environmental sustainability. It identifies prevention of unintended pregnancy as potential common ground for government policy-makers, environmentalists and family planning advocates. The health and other benefits of preventing unintended pregnancy are experienced most directly by individual women, men and their families. At the same time, preventing unwanted pregnancies usually results in smaller family size, an important factor in slowing population growth and, as a result, a source of broader benefits—including those affecting the environment—to society.

## 2. Population–environment interactions: population growth, consumption and human impact

It is estimated that half of the productivity of the Earth's biosystems has been diverted to human use, with concomitant depletion of our natural resources and impairment of the capacity of life-supporting ecosystems (Ehrlich & Ehrlich 1990; Green 1992; World Resources Institute 1998; United Nations Development Programme, United Nations Environment Programme, World Bank and World Resources Institute 2002; Wilson 2002; Brown 2004; Millennium Ecosystem Assessment 2005). Current size and continuing growth of the world's population adds to this environmental burden and, especially in places where growth is proceeding rapidly, will undermine the prospects for socio-economic development (Kendall 1992; National Academy of Sciences, National Academy of Engineering and Institute of Medicine 1993; Wilson 2002). The United Nations (UN) medium-variant population projection suggests that between 2008 and 2050, the world will have to accommodate 2.3 billion additional people. This growth, along with desperately needed advances in living standards for nearly 3 billion people in poverty will increase pressures on the environment (World Bank 2005; Population Division of the Department of Economic and Social Affairs of the United Nations Secretariat 2009).

The impact of humans on their environment is related to population size, *per capita* consumption and the environmental impact of the technology used to produce what is consumed. Although an oversimplification, this relationship has been represented by the ‘IPAT’ equation: ‘I (impact) = P (population) × A (affluence/consumption) × T (technology) (Ehrlich & Holdren 1971). It is an invalid assumption that environmental degradation grows in direct proportion to population size (assuming constant *per capita* consumption and modes of production). In particular, feedbacks, thresholds and synergies generally amplify risk, causing environmental degradation to grow disproportionally faster than growth in population size (Harte 2007).

Between 1950 and 2000, the world's population more than doubled, from 2.5 billion to 6.1 billion (Population Division of the Department of Economic and Social Affairs of the United Nations Secretariat 2007). At the same time, the gross world product expanded nearly sevenfold, from approximately $7 trillion to $46 trillion of annual output (Assadourian 2003). If *per capita* consumption were to grow at a modest rate of just 2 per cent annually, it would result in a fourfold increase in *per capita* consumption by 2075. Combined with a projected 52 per cent increase in population size over the same period (United Nations Department of Economic and Social Affairs Population Division 2004), this level of consumption could require economic production to increase sixfold. To achieve this without further degradation of important ecosystems presents a daunting challenge.

Although it received little attention, the recent UN-sponsored Millennium Ecosystem Assessment, conducted by more than 1300 experts in 95 countries, was an important and comprehensive study of the effects of ecosystem change on human health and well-being (Millennium Ecosystem Assessment 2005). It found that humans have changed ecosystems more rapidly and extensively over the past 50 years than during any other period, primarily to meet increasing demands for food, fresh water, timber, fibre and fuel. For instance, more land was converted to cropland in the 30 years after 1950 than in the 150 years between 1700 and 1850. It also estimated that 60 per cent of ecosystem services—the benefits people obtain from ecosystems—are being degraded or used unsustainably (Millennium Ecosystem Assessment 2005).

Other examples of ongoing environmental degradation include the following.

- Forests are dwindling: global forest cover has declined by 50 per cent since pre-agricultural times (United Nations Development Programme, United Nations Environment Programme, World Bank and World Resources Institute 2002). Rising use of forest products for paper, lumber and fuel is accelerating the process. Since the beginning of the twentieth century, 22 per cent of forest cover has been lost (Food and Agriculture Organization of the United Nations 2001), and in the Amazonian rainforest the decline is even steeper with a 20 per cent loss since 1970 (Kingstone 2005; Palermo 2005).
- Fisheries are endangered: with more than 2.9 billion people dependent on fish as a source of protein, 80 per cent of global fisheries have been over-fished or fished to their biological limit (Food and Agriculture Organization of the United Nations 2009).
- Cropland is shrinking because of soil erosion and desertification, and crop yields are threatened by rising temperatures and inadequate water supply (Brown 2006). Furthermore, between 1950 and 2007, the growth of world population has halved grainland per person from 0.23 ha to 0.10 ha (Brown 2008). Increasing diversion of grains to biodiesel, ethanol, and meat production additionally decreases supplies available for human consumption (Brown 2008).
- Water tables are falling as 15 countries containing half of the world's people, a total of 3.26 billion, are over pumping aquifers (Brown 2006). Depletion of aquifers threatens production of grains in the three largest producers—China, India and the USA—placing 175 million Indians and 120 million Chinese at risk of water shortage. Aquifer depletion threatens India with a 25 per cent decline in grain production during the next 50 years, when India's population is projected to increase by some 500 million, or 45 per cent (Worldwatch Institute 1999; Brown 2000, 2008). By 2025, three out of four people will face some degree of water scarcity (United Nations Environment Programme 2006).
- Global warming has doubled the percentage of the Earth's land area affected by drought from 15 per cent in 1970 to 30 per cent in 2002 (Brown 2006). The loss of glaciers that supply South America, China, India and many other countries with water threatens irrigation. Global warming has also reduced the snow pack that feeds irrigation and rivers in summer months in Afghanistan, Central Asia, Iran and the western USA (Brown 2008). Past record temperatures may become the norm by 2100, and a 1°C increase in mean temperatures will probably cause a 2.5–16% decline in crop yields, as already occurred in the 2003 European heat wave (Battisti & Naylor 2009). Warming is also slowing ocean circulation, causing more destructive storms, melting Arctic ice and bringing about rising sea levels (Brown 2006, 2008). A 10 m rise in sea level could displace more than 600 million people and flood large areas of cropland (McGranahan *et al*. 2007).

Brown has described the interaction between life-supporting ecosystems and population growth as follows: ‘as land and water become scarce, competition for these vital resources intensifies within societies, particularly between the wealthy and those who are poor and dispossessed. The shrinkage of life-supporting resources per person that comes with population growth is threatening to drop the living standards of millions of people below the survival level, leading to potentially unmanageable social tensions' (Brown 2008).

Living standards are already abysmally low for the more than 900 million people that the UN Food and Agriculture Organization estimates are chronically hungry. This situation is the worst among children and in Africa, where in 15 countries 35 per cent of the population is chronically hungry. It is estimated that malnutrition causes one in three children in developing countries to suffer physical and/or mental stunting (Black *et al*. 2008; Food and Agriculture Organization of the United Nations 2008).

The Sahelian region of Africa, which includes Darfur, is both rife with conflicts and has one of the world's fastest growing populations. In the northern Sahel, grassland is turning to desert, forcing herders southward into the farming areas. Declining rainfall and overgrazing are combining to destroy the grasslands (Brown 2008).

The population of sub-Saharan Africa is projected to increase from 809 million in 2008 to 1.7 billion in 2050 (Population Reference Bureau 2008*a*). Undoubtedly this will further intensify the competition for land, water, and food and increase the potential for conflict, social unrest and failed states.

## 3. World population growth

### (a) Demographic projections

Twentieth century advances in agriculture, public health and transportation, most markedly in the latter half of the century, yielded significant declines in death rates worldwide. Yet in many poor countries, the persistence of high birth rates resulted in rapid population growth. The century began with a world population of 1.6 billion and ended with 6.1 billion (McFalls 2007). World population has now reached 6.8 billion and is still increasing. Almost all current growth—about 1.5 million a week—occurs in developing countries and is most pronounced in Africa, a region projected to double in size, from the current 967 million to 1.93 billion in 2050 (Population Reference Bureau 2008*a*).

By contrast, with the exception of the USA, the 1.2 billion people living in wealthy developed countries are on the other side of a ‘demographic divide’—are characterized by birth rates so low that population decline and rapid ageing are almost inevitable.

**Figure 1.**
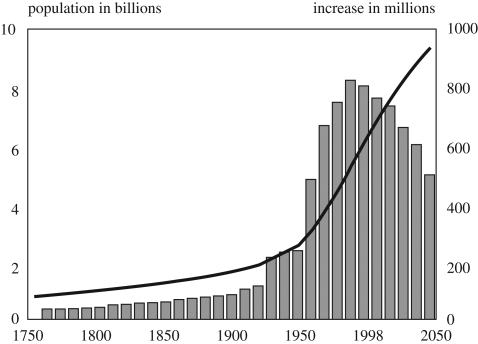
Estimated world population growth: 1750–2050 (McDevitt 1999). Black line, total world population; grey bars, population increase during the preceeding decade.

In 1950, the world's population was 2.6 billion, the average number of children per woman over her lifetime (total fertility rate or TFR) was 5.3, and annual population growth was 48 million (Population Division of the Department of Economic and Social Affairs of the United Nations Secretariat 2007). Widespread use of contraception has decreased the world's average TFR to 2.6 in 2008, but because death rates have also declined, about 78 million people are now added to the world's population each year (figure 1) (Population Division of the Department of Economic and Social Affairs of the United Nations Secretariat 2009). Developing countries are projected to account for 98 per cent of world population growth between 2007 and 2050 (Population Division of the Department of Economic and Social Affairs of the United Nations Secretariat 2009).

It took only 12 years, from 1987 to 1999, for world population to grow from five billion to six billion (United Nations Department of Economic and Social Affairs Population Division 1999). This is the shortest time ever to add one billion people—almost equivalent to the combined population of Europe and North America (Population Division of the Department of Economic and Social Affairs of the United Nations Secretariat 2007). Although the UN medium-variant projection suggests that the TFR worldwide will decline to 2.02 by 2050, population would still increase to 9.15 billion (Population Division of the Department of Economic and Social Affairs of the United Nations Secretariat 2009). The UN also produces alternative demographic scenarios: the low-variant, with fertility declining rapidly, projects a 2050 population size of 7.96 billion, whereas the high-variant projects a slower decline in fertility and a population of 10.46 billion (Population Division of the Department of Economic and Social Affairs of the United Nations Secretariat 2009).

If current family planning efforts are not strengthened and current levels of fertility were to remain unchanged, then world population is projected to reach 11.0 billion by 2050, rather than the 9.2 billion that is projected in the medium-variant (Population Division of the Department of Economic and Social Affairs of the United Nations Secretariat 2009).

Even if projected declines in fertility occur, the annual number of births worldwide is expected to remain high (McDevitt 1999). Previously high fertility rates have left many poor countries with large numbers of women of reproductive age; their numbers are projected to increase from 1.4 to 1.8 billion between 2005 and 2050 (Population Division of the Department of Economic and Social Affairs of the United Nations Secretariat 2007). As these increasing numbers of women have children, population size will increase even if fertility rates decline, a phenomenon known as population momentum. For example, although China's TFR has fallen below the replacement level of 2.1, the large number of couples of reproductive age has kept the country's population growing by about eight million annually (Population Reference Bureau 2008*a*).

Although fertility is low in Europe, Japan and a number of developing countries, high fertility persists in much of the developing world, ensuring that rapid population growth will continue. In 2008, TFR among the 4.2 billion people living in less-developed countries outside of China was estimated at 3.2 children, with an annual population growth rate of 1.8 per cent (Population Reference Bureau 2008*a*). At this rate, the population of these countries would double in just 39 years. Even taking projected declines in fertility in these regions into account, the number of people living in less-developed countries outside China is projected to increase by 60 per cent by 2050, to 6.6 billion (Population Reference Bureau 2008*a*).

### (b) Sources of world population growth

It might be assumed that future population growth will result primarily from the desire for large families in developing countries—but this is not the case. Bongaarts has estimated that most of the projected population increase in developing countries will result from population momentum (49%), followed by unwanted pregnancies (33%), and high desired family size (18%) (Bongaarts 1994, 2009).

The significance of unintended and unwanted pregnancies for population growth can be seen in the high proportion of pregnancies that are unplanned each year: out of 210 million pregnancies worldwide, 80 million (38%) are unplanned, and 42 million (20% of all pregnancies) end in abortion (Alan Guttmacher Institute 1999; Ahman & Shah 2007).

Reducing unintended pregnancy is the factor in continued population growth that is most amenable to programme and policy intervention. Organized family-planning programmes have a 40-year track record of success in helping hundreds of millions of couples choose the number and timing of their pregnancies.

Based on survey research, the Guttmacher Institute estimates that more than 200 million women in developing countries would like to delay their next pregnancy, or stop bearing children altogether, but rely on traditional, less-effective methods of contraception (64 million) or use no method (137 million) because they lack access to family-planning services; mistakenly believe that they are not at risk of getting pregnant because they were not fecund, were breastfeeding, or not having sex frequently; or face other barriers to using contraception (Singh *et al*. 2003). These barriers include cultural values that support high fertility, opposition to use of contraception by family members and others, fears about health risks or side effects of contraception, and access to only a few contraceptive options (Carr & Khan 2004; Sedgh *et al*. 2007).

## 4. US population growth

### (a) US demography

The US population size is important because of the country's high level of consumption and because, in contrast to almost all other developed countries, the US is experiencing rapid population growth (Perry & Mackun 2001). Now the world's third largest country, the US is projected to grow from 305 million in 2008 to 356 million in 2025, and to 438 million by 2050 (Population Reference Bureau 2008*a*). As a result, the US has a disproportionately large environmental impact.

### (b) Sources of US population growth

Natural increase (births exceeding deaths) accounts for 58 per cent of population growth in the USA, with 4.15 million births and 2.46 million deaths in 2006; net immigration of 1.2 million people accounts for the remaining 42 per cent of the annual growth of 2.9 million people (Kent & Mather 2002; US Census Bureau 2006).

Unintended pregnancy is a major contributing factor to the relatively high birth rate in the US. Of 6.4 million pregnancies in 2001—the most recent year for which data is available—almost half (3.1 million) were unintended (as were 82% of the 811 000 annual teenage pregnancies). These pregnancies resulted in 1.1 million miscarriages, 1.3 million abortions, and four million births, of which 1.4 million were unintended (Finer & Henshaw 2006). Without these 1.4 million unintended births, the natural increase of the US population would be about 300 000 per year, less than 20 per cent of current natural increase. In other words, unintended pregnancy accounts for roughly half of the current increase of 2.9 million people to the US population each year (US Census Bureau and Population Division 2006).

The contribution of immigration to US population growth is also important. Between 2000 and 2004, 4.3 million immigrants, including an estimated two million undocumented immigrants, arrived in the country (Camarota 2004). Passel estimates that there are 11.5–12 million undocumented immigrants in the US (Passel 2006). There is also an important relationship between immigration and natural increase: nearly one-quarter of babies born in 2002 had a foreign-born mother, an increase from six per cent in 1970 (Camarota 2005).

### (c) US environmental trends

The US already has the world's largest ‘environmental footprint’, i.e. the greatest *per capita* consumption compared with the productivity of US ecosystems and natural resources. A recent study has documented the deleterious synergies between US population growth and the rapidly increasing *per capita* consumption of resources (Markham & Steinzor 2006). Among the report's findings are the following.

- Land-use: US land is converted for development at about twice the rate of population growth. The most predominant form of land use change is ‘sprawl’—low-density development spread into suburban and rural areas, with high vehicle use and new roads, shops and other infrastructures.
- Water: Use by Americans is three times the world's average. More than half of the nation's wetlands are gone and more than 40 per cent of the nation's rivers, lakes and estuaries are too polluted for fishing and swimming.
- Biodiversity: About 6700 known plant and animal species are considered at risk of extinction, mainly (85%) from habitat loss and alteration attributed primarily to human activity. About one-third of America's freshwater animal species are at risk.
- Forests: The US is the world's largest consumer of forest products, increasing by 50 per cent in the past 40 years.
- Fisheries and aquatic resources: Thirty per cent of assessed fish populations in the US coastal waters are either over fished or fished unsustainably. A high proportion of fish caught in US rivers, lakes and coastal areas present health risks because of contamination by mercury and other pollutants.
- Agriculture: Nearly 3000 acres of US farmland are lost every day to development.
- Energy: With only 5 per cent of the global population, the US consumes almost 25 per cent of the world's energy. America has the highest oil consumption worldwide, and is projected to use 43 per cent more oil than the current levels by 2025. Transportation is the nation's fastest growing energy use sector.
- Climate change: The US is the second largest carbon dioxide greenhouse gas emitter in the world, accounting for nearly one-quarter of all global emissions. The nation's average temperature increase during the next 100 years is projected to be 2.8–5°C. Sea level rise and more severe weather events that will impact coastal areas are predicted, particularly in the US Mid-Atlantic and Gulf Coasts.
- Waste: Each American produces about five pounds of rubbish daily, five times the average amount in developing countries.

## 5. Policies to address population growth

### (a) International agreements

The 1994 International Conference on Population and Development (ICPD) set a broad agenda for population work (UNFPA 1995). In addition to the provision of basic family planning and other reproductive health services, it emphasized poverty eradication, women's empowerment, gender equity, human rights, environmental protection, male responsibility in sexual behaviour and family welfare, adolescent reproductive health and safe abortion (Germain & Kyte 1995).

In 2000, the UN Summit issued a Millennium Declaration and shortly thereafter Millennium Development Goals (MDGs), though these did not explicitly address the broad array of reproductive health issues from the ICPD or call for access to family planning. However, in 2005, the UN Millennium Project's report, ‘*Investing in Development: A Practical Plan to Achieve the MDGs*’, argued that expanding access to sexual and reproductive health information and services is part of a core group of necessary, affordable and effective actions that can speed progress towards achieving the larger MDGs (UN Millennium Project 2005). The 2005 UN World Summit took the additional step of explicitly incorporating the ICPD goal of universal access to reproductive health as a target under the fifth MDG on improving maternal health. This action was approved by the UN General Assembly in 2006.

### (b) Strengthening family planning deserves high priority

Reproductive health problems related to pregnancy, childbirth and STIs including HIV/AIDS represent nearly one-fifth (18%) of the global burden of disease (GBD).^1^ For women of reproductive age, such problems account for one-third of the disease burden, and an even higher share among women in developing countries (Vlassoff *et al*. 2004). Thus, it is easy to see why improving reproductive health—especially as it relates to avoiding unintended births, ensuring safe childbirth and HIV/AIDS prevention, treatment and care—is intrinsically desirable. Family planning, in particular, is not only important to improving reproductive health, but also to enabling women and men to choose the number and timing of childbirth—a basic human right. Provision of family-planning services is also the most direct intervention to slow population growth and assist environmental preservation (Potts 1997; Vlassoff *et al*. 2004).

According to Potts: ‘all societies with unconstrained access to fertility regulation, including abortion, experience a rapid decline to replacement levels of fertility, and often lower’ (Potts 1997). Better contraceptives and the establishment of organized family-planning programmes have successfully met the demand for small families and decreased fertility.

- Between 1960 and 2008, contraceptive prevalence in less-developed countries increased from 9 per cent (about 30 million users) to 62 per cent (about 630 million users) among married women of reproductive age (Population Division of the Department of Economic and Social Affairs of the United Nations Secretariat 2005; United Nations Department of Economic and Social Affairs Population Division 2007; Population Reference Bureau 2008*a*,*b*).
- During the same time period, the TFR in developing countries declined by more than half, from 6.0 to 2.8 (Population Division of the Department of Economic and Social Affairs of the United Nations Secretariat 2005; Population Reference Bureau 2008*a*).

The importance of abortion in child-bearing choices is seldom recognized. Given the high unmet need for family planning and the high failure rates of existing methods of contraception, access to safe abortion is necessary for women to fully control their fertility. It is estimated that about 12 per cent of pregnancies end in abortion in Africa, 23 per cent in Latin America and the Caribbean, and 30 per cent in East Asia (including China and Japan, where abortion is legal) (Alan Guttmacher Institute 1999). Without the 42 million abortions worldwide each year, population growth would be much more rapid (Alan Guttmacher Institute 1999; Ahman & Shah 2007). Unfortunately, in 2003 about 20 million of these abortions were medically unsafe and caused 13 per cent of all maternal deaths—about 67 000 of 529 000 deaths annually. An estimated 97 per cent of unsafe abortions occur in developing countries where, on average, each woman will experience one unsafe abortion during her child-bearing years. This is one reason why the ICPD identified unsafe abortion as a major public health concern (Crane & Hord Smith 2006; Ahman & Shah 2007).

The impact of organized family-planning programmes on population growth is clear: such programmes were responsible for at least 40 per cent of the fertility decline in developing countries from the 1960s through to the 1980s (Vlassoff *et al*. 2004). At the same time, fewer pregnancies, which are appropriately spaced, result in less exposure to the risks associated with pregnancy and childbirth. Indeed, use of family planning could prevent at least one-quarter of maternal deaths in developing countries (Levine *et al*. 2006). In Pakistan, for example, preventing all births after the fifth would reduce maternal deaths by half (Eastwood & Lipton 2006).

## 6. Family-planning programmes slow population growth: three case studies

The experiences of Thailand, Iran and California demonstrate how publicly supported family-planning programmes can successfully curb rapid population growth.

### (a) Thailand: slowing population growth through innovative methods

Thailand's government launched its population programme in 1970, making a broad array of contraceptives including injectable and oral contraceptives available without a prescription. Contraceptives were distributed by nurses, midwives and even shopkeepers within communities (Rosenfield *et al*. 1971). Programme results include the following.

- By the late 1980s, Thailand's TFR had dropped below replacement level to fewer than 2 births per woman (bpw) (compared with about 7 bpw just two decades earlier) and currently remains low at 1.7 (Hirschman *et al*. 1994; Population Reference Bureau 2008*a*).
- Cost–benefit analysis estimates that Thailand's programme will have prevented 16.1 million births between 1972 and 2010, saving the government $11.8 billion in social service costs, or $16 for every dollar invested in the programme (Chao & Allen 1984).

### (b) Iran: improving family planning with political and religious support

Recognizing an impending imbalance between available natural resources and population size, the Iranian government, with the support of Muslim religious leaders, restored its national family-planning programme in 1989 with the following results (Hoodfar & Assadpour 2000; Larsen 2001).

- Between 1976 and 1997, the proportion of married women of reproductive age using contraception increased from 37 to 73 per cent (Aghajanian & Merhyar 1999).
- After reaching 6.8 in 1984, the TFR dropped from 5.5 in 1988 to 2.8 in 1996 and is currently at the replacement level of 2.1 bpw (Abbasi-Shavazi 2002; Population Reference Bureau 2008*a*).

### (c) US: a cost-effective programme for California's low-income residents

Low-income women represent 40 per cent of women in California of reproductive age and account for nearly two-thirds of births in the state (Braveman *et al*. 1999). An estimated 1.7 million women in California are in need of publicly funded family-planning services (Chabot *et al*. 2006). In 1997, the California legislature initiated the Family PACT (Planning, Access, Care and Treatment) Programme to provide clinical family planning and reproductive health services at no cost to low-income residents with the following results.

- During the programme's first 5 years, the number of clients served more than doubled—from 750 000 to 1.55 million (Bixby Center for Reproductive Health Research & Policy 2005).
- The contraceptive services provided by Family PACT in 2002 averted an estimated 205 000 unintended pregnancies (Foster *et al*. 2006). Every dollar spent on Family PACT avoided public expenditures—for medical care, income support and social services for women and their children—that would have cost $2.76 over 2 years and $5.33 over 5 years (Brindis *et al*. 2005). An investment of $403.8 million in 2002 therefore saved $1.1 billion over 2 years and saved $2.2 billion over 5 years.

The experience from these diverse settings shows that impressive declines in fertility and population growth are possible in a short period of time through implementation of well-managed, fully voluntary family-planning programmes that meet the needs of individuals and families. Similar programmes could—at relatively low cost—yield a substantial impact on population growth worldwide and help to alleviate the increasing burden it places on the environment.

## 7. International population challenges

### (a) Unmet need

Of the 201 million women in developing countries with an unmet need for contraception, 64 million rely on high-failure, traditional methods, often owing to lack of access to modern methods (Singh *et al*. 2003). A survey of 89 developing countries found that only 57 per cent of couples had reasonable access to five modern contraceptive methods (pills, intrauterine devices, condoms, and male and female sterilization) (Ross & Stover 2001). In 35 countries including Afghanistan, the Democratic Republic of Congo, Ethiopia and Nigeria, modern contraceptive use among married women is less than 15 per cent (Population Reference Bureau 2008*b*). Unfortunately, many countries, including Kenya, Nigeria and Tanzania, are experiencing contraceptive shortfalls resulting from increasing demand for services and decreasing donor funds to support them (Finkle 2003).

### (b) Discontinuation and failure rates

Many couples do not use contraception even when it is available because of concerns about side effects or unrealistic fears that contraceptives are dangerous. Other couples are limited to a modern method that is unsuitable to their circumstances, leading to high discontinuation rates. In some countries, particularly those in which contraceptive use is already high, efforts to improve contraceptive continuation may therefore be more appropriate than efforts to reduce unmet need (Westoff & Bankole 2000). Modern contraceptives also fail frequently, mainly because of incorrect use; even for the most effective methods, such as the pill, a failure rate of 8 per cent is typical (Trussell 2004). A study of 15 diverse developing countries found that contraceptive failure increased the TFR by 4–29% (Blanc *et al*. 2001).

### (c) Unintended pregnancies

Of the 5.5 billion people living in developing countries in 2008, one in four (1.4 billion) were women of reproductive age (Singh *et al*. 2003; Population Reference Bureau 2008*a*,*b*). More than half were at risk for unintended pregnancy because they were sexually active, able to become pregnant, and had either completed their childbearing or did not want children in the near future (Singh *et al*. 2003). Nevertheless, according to Demographic and Health Survey estimates for 51 developing countries, almost one-half of pregnancies and one-third of births in the developing world are ill-timed or unwanted (Westoff 2001).

### (d) Reaching adolescents

As a result of high fertility during the last four decades, there are more young people in the world than ever before—more than one billion young women and men between ages 15 and 24 (Population Division of the Department of Economic and Social Affairs of the United Nations Secretariat 2005). These young people are reaching their peak childbearing years and thus will shape the world's demographic destiny. In many parts of the developing world, a high percentage of births occur among women younger than age 20, underscoring the importance of efforts to postpone early childbearing (Ashford & Clifton 2005).

### (e) Abortion

Given the high unmet need and failure rates for the existing methods of contraception, access to safe abortion is necessary for women to fully control their fertility. Also, without the 42 million abortions worldwide each year, population growth would be much more rapid (Ahman & Shah 2007).

### (f) Assisting the transition to low fertility in developing countries

Unlike the long-term demographic transitions in Europe and the USA, which involved minimal government intervention, developing countries require substantial government and donor investment to hasten their demographic transitions. This investment includes financing of resources that otherwise are not available (e.g. contraceptive supplies and training) and supporting policies that indirectly affect fertility rates (e.g. promoting female education) (Cross *et al*. 2002). New development priorities, such as general debt relief and support of broad sectors rather than specific programmes have diverted funds from family planning. These new approaches to development assistance, combined with conservative policies that cater to religious groups and anti-abortion activists, threaten government-sponsored population programmes both in the developing countries and in donor nations.

### (g) Meeting the growing need for financial resources

Increased financial resources are needed not only to maintain current levels of contraceptive use, but also to meet the unmet and growing demand for family planning. According to the UN Population Division's medium-variant projection, the number of women aged 15–49 in less-developed regions is projected to increase from 1.38 billion in 2005 to 1.75 billion in 2030 (Population Division of the Department of Economic and Social Affairs of the United Nations Secretariat 2007). This increase of 370 million women, most of whom will need family-planning services, is in addition to the 200 million women in developing countries who already lack quality family-planning services. Even if the proportion of couples using contraceptives were to remain level between 2000 and 2015, because of population growth, developing countries would still have to serve 125 million additional couples (Ethelston *et al*. 2004). However, the global community has failed to provide the funds to satisfy the current unmet demand for family planning, let alone fulfil the ICPD goal of universal access to family planning, maternal health and other basic reproductive health services.

#### (i) Funding targets

Various estimates of funding needs are shown in table 1. In 1994, the ICPD estimated the cost of programmes for family planning, safe childbirth and STI/HIV prevention at $18.5 billion for 2005 ($27.2 billion in 2008 dollars when adjusted for inflation), of which two-thirds was to come from developing countries and one-third from donor countries (UNFPA 1995). These ICPD estimates included all delivery system costs in the figure for family planning. The estimate for reproductive health only included funds for safe childbirth services and the estimate for STI/HIV/AIDS only included preventive services.

**Table 1.** Funding targets for family planning (FP), reproductive health (RH), and STI/HIV/AIDS programmes in developing countries (in $ billions).

activity	original 1994 ICPD target for 2005 (UNFPA 1995)	inflation-adjusted ICPD target for 2005^a^	2003 estimate by Guttmacher Institute and UNFPA (Singh *et al*. 2003)	revision of ICPD 2005 FP and RH target by Speidel and 2006 HIV/AIDS target by UNAIDS (Speidel 2005; UNAIDS 2005)	2008 target estimate by authors based on Stover (Gillespie *et al*. 2009)^b^	2009 target for HIV/AIDS by UNAIDS in 2007 (UNAIDS 2007)	revision of ICPD 2009 FP, RH and STI/HIV/AIDS target by UNFPA and UNAIDS in 2009 (United Nations Economic and Social Council 2009)	revision of ICPD 2015 FP, RH and STI/HIV/AIDS target by UNFPA and UNAIDS in 2009 (United Nations Economic and Social Council 2009)
	(1993 $)	(2008 $)	(2008 $)	(2008 $)	(2008 $)	(2008 $)	(2009 $)	(2009 $)
FP direct costs							2.3	4.1
FP (including all delivery system costs)	11.5	16.9	12.7	16.9	14.8			
RH (maternity and additional services)	5.4	7.9		16.3			6.1	18.0
programme- and system-related costs for FP and RH							15.0	10.9
total sexual/RH/FP	16.9	24.8		33.2			23.5	33.0
STI/HIV/AIDS (prevention)	1.4	2.1						
STI/HIV/AIDS (prevention, treatment, care and support)				15.7		23.1	24.0	36.2
research, data collection, policy analysis	0.20	0.29		0.29			1.6	0.6
total FP, RH, STI/HIV/AIDS research, data and policy	18.5	27.2		49.2			49.0	69.8
donor country share (1/3, 2/3 AIDS)	6.1	9.0		21.6			24.3	35.3
developing country share (2/3, 1/3 AIDS)	12.4	18.2		27.6			24.7	34.5

^a^Adjustment for inflation. Adapted from http://data.bls.gov/cgi-bin/cpicalc.pl (accessed 14 February 2009).

^b^Assumes 70 per cent of 1.443 billion women of reproductive age are married or in union, contraception is used by 85 per cent, and $17.24 is the annual cost per user. Therefore the total for FP is $14.8 billion.

The Guttmacher Institute and the UNFPA revised the cost estimates for family planning in 2003, calling for $12.7 billion (in 2008 dollars) for family planning, a figure that includes health system costs (Singh *et al*. 2003).

Revised cost estimates by Speidel in 2005 called for $15.9 billion in annual spending for family planning and basic research and $15 billion for reproductive health; the same year UNAIDS called for $14.9 billion for the full range of HIV/AIDS prevention, treatment, care and support services. UNAIDS also called on donors to provide two-thirds of the total for STI/HIV/AIDS programmes ($10 billion) (Speidel 2005; UNAIDS 2005).

Adjustments for inflation and the increased price tag for a broader array of reproductive health and STI/HIV/AIDS services bring the revised annual funding target for 2005 to $45.8 billion ($49.2 billion in 2008 dollars) rather than the original $18.5 billion.

In 2007, UNAIDS revised their target, calling for donor and developing country expenditures of $22.5 billion for HIV/AIDS in 2009, reaching $49.5 billion annually by 2015 (UNAIDS 2007).

Stover uses another approach, estimating that for each client family-planning services average $17.24 per year. If it is assumed that in 2008 there were 1.4 billion women of reproductive age; 70 per cent of these women were married or in union; and ideally 85 per cent would use contraception, then the family planning funding target would become $14.8 billion (Gillespie *et al*. 2009).

In 2009, the UNFPA, working with the World Health Organization and a group of expert advisors, conducted a new analysis of funding needs for each of the ICPD categories. These estimates grouped all programme- and system-related costs for family planning and maternal health into a single category, with estimated funding needs of $15 billion in 2009. Funds for direct family-planning costs were estimated at an additional $2.3 billion and for direct maternal health costs were estimated at an additional $6.1 billion, for a total of $23.5 billion. With the inclusion of $1.55 billion for research, data collection, and policy activities and $23.98 billion for HIV/AIDS, the revised ICPD annual cost estimates total $48.98 billion for 2009 (United Nations Economic and Social Council 2009).

Taken together these estimates suggest that annually about $15 billion is needed for family planning and related service delivery systems, and close to $50 billion is needed for the comprehensive ICPD programme that includes family planning, other reproductive health services, research, training, data collection and addressing HIV/AIDS.

#### (ii) Progress in meeting funding goals

The revised UNFPA target of nearly $49 billion called for donors to provide about $24.3 billion and the developing countries about $24.7 billion (table 2). These targets can be compared with provisional figures for 2007 of $8.13 billion being provided by donors, $7.7 billion by developing country governments and non-government organizations, and $10.8 billion spent by consumers for a total of $18.5 billion from domestic sources (United Nations Economic and Social Council 2009).

**Table 2.** Revised 2009 ICPD funding targets for family planning, reproductive health and HIV/AIDS compared with provisional 2007 population assistance and domestic expenditures (in $ billions and per cent). Donor share of total is one-third, except share for STI/HIV/AIDS, which is two-thirds.

	2007 provisional expenditures (2007 $)	revised UNFPA ICPD target for 2009 (2009 $)	% of target
donor share	8.13	24.65	33
developing country share	18.46	24.33	76
total	26.59	48.98	54

It should be recognized that the apparent good performance of developing countries with regard to domestic expenditures shown in table 2 is misleading in many instances. The generous support of reproductive health programmes in a few large countries, including China, India and Indonesia, obscures the dismal performance of the governments of many other countries, particularly those in Africa. Furthermore, the estimates of consumer spending, the source of more than half of domestic expenditures, are imprecise, and the quality of services offered is likely to be sub-standard in many settings.

An analysis of donor funding by area of activity shows great divergence in priorities. The most recent increases in donor outlays for population assistance have been for HIV/AIDS, while donor funds specifically allocated for family planning have decreased during the past 12 years. Between 1995 and 2007, the most recent year with provisional data (United Nations Economic and Social Council 2005; United Nations Economic and Social Council 2009):

- funding for STI/HIV/AIDS activities has increased from 9 to 75 per cent of the $8.13 billion provisional total for population assistance;
- the proportion of funding for basic reproductive health services has changed a little, from 18 to 17 per cent;
- funding explicitly for family-planning services decreased, from 55 per cent to just 5 per cent; however, some family planning is now funded from within the reproductive health category; and
- funding for family-planning activities decreased in absolute dollar amounts from $723 million to $407 million.

In addition, funding for basic research activities accounted for only 3 per cent of expenditures in 2007.

If the revised targets for donor funding in 2009 are compared with the provisional 2007 donor contributions, family planning plus reproductive health assistance reached only 16 to 23 per cent of the two annual outlays targets shown in table 3. Donor assistance for STI/HIV/AIDS in 2007 reached 38 per cent of the UNFPA/UNAIDS target for 2009.

**Table 3.** 2008 and 2009 ICPD funding category targets for *donors* for family planning (FP), reproductive health (RH) and STI/HIV/AIDS services compared with estimated 2007 donor population assistance by category targets (in $ billions and per cent). Donor targets were assumed to be one-third of totals needed except for STI/HIV/AIDS targets, where donor share is assumed to be two-thirds.

expenditure category	2007 donor expenditures (provisional) (United Nations Economic and Social Council 2009)	Speidel/UNAIDS revised donor target for 2008 (Speidel 2005; UNAIDS 2005)		UNFPA revised ICPD donor target for 2009 (United Nations Economic and Social Council 2009)	
	(2007 $)	(2008 $)	% of target	(2008 $)	% of target
FP	0.407	5.63	7		
RH	1.38	5.43	25		
FP and RH	1.79	11.06	16	7.82	23
STI/HIV/AIDS	6.10	10.5	58	16.0	38
basic research	0.244	0.100	244	0.517	47
total	8.13	21.7	37	24.3	33

Clearly, population programme assistance provided by donors, particularly for family planning, is insufficient to achieve the goals of the ICPD and the MDGs. Many developing country governments, especially in the least developed countries, provide almost no domestic funds for population programmes and rely almost entirely on development assistance. Since more funds are available to address HIV/AIDS activities, less priority is given to other health needs including family planning, maternal health and other reproductive health activities.

## 8. US population challenges

Despite progress in states such as California, as well as nationwide reductions in teenage pregnancy and birth rates, unintended pregnancy remains a major problem for the US and significantly contributes to population growth in the country. Challenges to addressing unintended pregnancy include the following.

- Comprehensive sexuality education—which could help prevent pregnancy and is favoured by a majority of parents and educational experts—has been replaced by ‘abstinence unless married’ programmes, which since 1982 have received major increases in federal funding with total expenditures of $1.5 billion through 2008 (National Family Planning and Reproductive Health Association 2009). Given that 62 per cent of male and 70 per cent of female teenagers become sexually active, these programmes leave many teenagers unprepared to make informed decisions regarding their sexual and reproductive health (Mosher *et al*. 2005).
- Efforts are also underway to eliminate confidentiality and requiring parental consent for teenagers seeking family-planning services, even though research shows that if confidentiality were lost, teenagers would stop attending clinics, but would not stop having sex (Jones *et al*. 2005).
- Public funding for family-planning services is not keeping pace with demands. At $307.5 million in 2009, inflation-adjusted funding for Title X, the nation's only distinct, federally funded family planning programme, has declined by more than half since 1980 (Alan Guttmacher Institute 2000).

A recent study estimates the annual cost of family planning per client at between $124 and $487 with a mid-range estimate of $203 (Frost *et al*. 2006). In 2005, California's Family PACT Programme spent $236 per client. With 17 million US women reliant on public funding for contraceptive services, an annual expenditure of about $3.5 billion is needed (Sonfield 2003). This can be compared with public outlays of $1.85 billion for contraceptive services in 2006—about one-half of the total needed (Sonfield & Gold 2005).

Through the Medicaid ‘waiver’ programme, the federal government pays up to 90 per cent of states' family planning programme costs for expanded eligibility for family-planning services. Yet, only 27 states have taken advantage of this option, and stringent income and other eligibility requirements for the programme exclude a significant proportion of low-income women and men in need of services (Alan Guttmacher Institute 2005). To address US family planning needs, Title X funding must be markedly increased, and all states should capitalize on the substantial health and fiscal resources available through Medicaid waivers. If the need for waivers were eliminated, and coverage of family planning through Medicaid were required of all states, an additional 3.5 million women a year would be eligible for services; an estimated 500 000 unintended pregnancies would be averted; and states and federal government would save about $1.5 billion a year (Frost *et al*. 2006).

## 9. Addressing population: why the neglect?

Reasons why attention to population issues, and especially abortion, has waned include:

- the success of family planning and attention to declining birth rates;
- UN projections of population growth ending around 2050;
- low fertility in most developed (and a few developing) countries;
- criticism at the ICPD of past population work and advocacy for a less focused, ‘ICPD new paradigm’ of reproductive health;
- the influence of vocal anti-abortion activists, conservative religious leaders and conservative think tanks;
- shifting attention and resources—both human and financial—away from population growth in response to the AIDS crisis; and
- ‘donor fatigue’—the tendency to move on to emerging new issues at the expense of continuing problems.

As population issues in general have received decreased attention, so has the interaction between population growth and environmental sustainability. This problem is compounded by the fact that the relationship between population and the environment is a complex and often controversial one. Wilson and Kehoe (2000) have summarized the reasons of why environmental organizations have failed to advocate around population:

… environmental organizations have found it difficult to frame population issues in ways that appeal to the concerns of American environmentalists yet avoid causing internal dissension, raising ethical dilemmas or implicating politically problematic issues. Factors inhibiting unequivocal commitment to population by environmental groups have included: difficulty in applying environmental groups' legal or scientific expertise to the global population issue; the perception that opportunities for success on this issue are limited; domestic controversies, especially debates over the extent to which the groups should address U.S. immigration policies; and sensitivities surrounding North/South dynamics in international relations and the moral dilemmas stemming from America's high rate of consumption relative to the rest of the world (Wilson & Kehoe 2000).

Academic scholarship infrequently addresses the population–environment relationship. Environmental scientists often use UN statistics for their population projections, but may not recognize that these could be too optimistic, especially if population programmes continue to be neglected (Bongaarts *et al*. 1990; Bongaarts 2005). There is also a tendency to focus on the ‘good news’ of declining birth rates, rather than the continuing large increases in world population size.

Understandably, scientists concerned about the broad array of environmental issues look within their own disciplines for solutions, often through development and implementation of new, eco-friendly technology. Similarly, some influential economists, business leaders and conservative government policy-makers downplay the importance of the population–environment connection in favour of market-based economies and scientific progress as solutions. Also, the cost-effectiveness of investing in population and family-planning programmes compared with many other investments to protect the environment is not well understood (Bongaarts 1994; Bongaarts *et al*. 1997).

Some environmental activists and organizations do have a long history of concern about population. They include the National Audubon Society, the Sierra Club, the National Wildlife Federation, the Worldwatch Institute, the Earth Policy Institute, the Izaak Walton League of America and the World Wildlife Fund. Other organizations have decreased their emphasis on the link between population and the environment in response to concerns about population programmes that have focused narrowly on reducing growth rates, and programmes with coercive components such as China's ‘one-child’ policy. Finally, there are groups that will not tackle population issues because they consider the subject to be too controversial for their boards, their members, their donors or those they seek to influence.

A number of influential groups that focus more broadly on women's health and welfare have adopted the ideological stand that ‘numbers do not matter’. Since concern about the environment inevitably must consider population numbers, addressing population–environment relationships has been given little attention by these reproductive health advocates. Their prominent presence at the ICPD strengthened the conference's emphasis on broad policies that give high priority to women's education, welfare and health. Clearly, these issues deserve increased attention, but to some extent this emphasis resulted in neglect of population, family planning and environmental issues (McIntosh & Finkle 1995).

Conservative religious activists, who are often opposed to contraception as well as abortion, indirectly influence environmental policy by denigrating concerns about population issues. Family planning and reproductive rights are often at odds with religious views on family structure and morality, especially regarding the sexual behaviour of teenagers and abortion. Thus, some religious groups will not support a rationale for environmental preservation that requires strong family-planning policies and programmes.

Finally, many governments avoid attention to links between population and environment. At the Earth Summit in Rio in 1992, and in the formulation of the MDGs, population was deemed too controversial to address explicitly (Crossette 2004). Increasing conservatism on the part of many developed country governments, new concerns about terrorism and war, and the continued emphasis on US political discourse on mainstream issues such as the economy, crime, healthcare, education and taxes, leaves little room for a national debate about population and the environment.

## 10. Action agendas to address population

Governments, non-government organizations and the general public must mobilize the political will and resources to address the twin challenges of rapid population growth and environmental degradation. Current action is inadequate.

Environmental organizations can make an important contribution to this cause by educating their membership, policy-makers and the public about the need for global action to improve access to family planning both in the US and worldwide. Organizations concerned about the environment, population and/or economic development should pool resources to build and mobilize a base of grassroots activists to advocate for improved family planning and reproductive health policies and programmes, and to help raise public awareness about the links between population and environment.

An action agenda for developing countries should seek to implement the following.

- *Improve the policy environment*: both donors and developing country governments place inappropriate policy restrictions on access to family planning information and services. These restrictions are particularly onerous for unmarried and/or adolescent women, as well as those in need of abortion services.
- *Strengthen family planning and related reproductive health services*: in too many countries, these services are of low quality—underfunded, understaffed, lacking a broad variety of modern contraceptives—or are entirely unavailable. In settings with a high prevalence of HIV/AIDS or where risk of HIV infection is high, family planning and HIV/AIDS programmes should be integrated. Information about human sexuality, family planning and reproductive health must also be provided as an essential component of service-delivery programmes (Berer 2004).
- *Increase human, commodity, and financial resources*: about $24 billion annually is needed for family planning and reproductive health (excluding HIV/AIDS) programmes in developing countries. Unfortunately, neither developing country governments nor donors are anywhere near meeting the financial needs of these programmes. Of particular concern is the shortfall of contraceptive supplies to meet increasing demand. Their purchase from sources in developed countries can require developing countries to use scarce hard currency, making them difficult to obtain and appropriate for donors to provide as a part of their foreign assistance package. Equally important is the lack of trained personnel to manage and operate family planning and reproductive health programmes.
- *Support safe abortion services*: worldwide, an estimated 42 million women have abortions each year (Ahman & Shah 2007). However, the legality of abortion varies by country: 61 per cent of women live in countries with ‘liberal’ abortion laws, while 25 per cent reside in a country where abortion is illegal or allowed only to save a woman's life, and 14 per cent live in countries where abortion is only permitted to protect a woman's physical or mental health or to save her life (Alan Guttmacher Institute 1999). Yet, support for safe abortion services on the part of donors and governments remains controversial and varies according to domestic politics. Nevertheless, given the ongoing need for abortion and the high mortality associated with unsafe procedures, ensuring a supportive legal and policy environment as well as the provision of safe abortion services should be a priority.
- *Invest in research*: improved contraceptive and abortion technologies are needed to overcome issues relating to effectiveness, safety, cost, acceptability and side effects that hamper use of current methods. Demographic research and operations research to improve family planning and reproductive health delivery systems is also needed.
- *Implement development programmes that help slow population growth*: general economic development, especially investments in improving human capital, gender equality and education—especially for girls—all contribute to reduced fertility. The population momentum resulting from the young age structures in many developing countries can be partially offset by increasing the age of first childbirth as well as the intervals between births. There is a need for increased development assistance from the estimated 2007 figure of $103.5 billion to more than $250 billion annually—the target level to meet the agreed-upon standard of 0.7 per cent of gross national product (Ethelston *et al*. 2004; Organisation for Economic Co-operation and Development Assistance Committee 2008).^2^

### (a) An action agenda for the US

A combination of anti-abortion activism and increasing social conservatism in local, state and national governments has hampered progress in US policies and programmes relating to population, family planning, and reproductive health and rights. This has translated into poorly funded family-planning programmes and restrictive policies relating to abortion, sexuality education and reproductive health services. In addition, immigration law and policy are out of step with the reality (especially with regard to undocumented immigrants), as low cost labour is often welcomed, and the legal status of employees is overlooked.

Early indications suggest that the new Obama administration and the Democratic majority in Congress will bring increased attention to family planning and reproductive health. An agenda for US advocacy and programmes should seek to do the following.

- *Initiate research on and discussion about population growth and its implications for the environment*: there is a need for increased recognition of the environmental and other costs associated with continuing rapid population growth in the US. Considering that the Census Bureau's medium-variant projection estimates nearly 600 million Americans by 2100, and the high variant suggests as many as 1 billion (Hollmann *et al*. 2000), it is time for the US to adopt an official population policy.
- *Strengthen family planning policies and programmes*: all reproductive health policies and programmes should be based on scientific evidence, rather than ideology. The Title X programme is in need of increased funding, and Medicaid must maintain the current 90/10 federal/state split of funding. In addition, the ‘waiver’ for Medicaid funding should be eliminated, and all states should be required to provide family-planning services for low-income women and men. Minimizing unintended pregnancy would substantially slow US population growth and does not require addressing more controversial population-related issues.
- *Increase access to abortion*: multiple policy restrictions on abortion—such as mandatory waiting periods and parental involvement laws—should be eliminated. There is also an urgent need to increase the number of medical practitioners offering abortion services, given that 87 per cent of US counties lack an abortion provider (Finer & Henshaw 2003). Provision of free or low-cost abortion services for those who cannot afford them would ensure access for women of all socio-economic levels.
- *Support educational and service programmes for young people*: adolescents should receive education that addresses human sexuality, contraception and abortion, including, but not limited to, abstinence. Teenage-friendly services that include abortion are a key contributor to low teenager birth rates in European countries and declining rates in the US.
- *Seek new solutions to immigration*: despite the complexity and controversy of the issue, immigration requires attention. Although the topic is controversial, there are some approaches to it that are not; for example, there should be little disagreement about efforts to improve economic opportunity and family planning in sending countries to diminish the economic and population-related motivation for emigration.

In summary, environmental advocates and conservation programme planners generally understand the importance of population issues, but have often given them a low priority. Reasons include lack of scientific expertise, the belief that tackling population issues is too controversial or unlikely to yield success, and a perceived absence of moral standing given the disproportionately high rates of consumption in developed countries. Prevention of unintended pregnancy is a strategy that, for most of the public, is not controversial, yet can have a substantial impact on reducing population growth and the concomitant pressures it places on the environment. The successful family-planning programmes found in many settings, as diverse as Thailand, Iran and California, show that such programmes are desired, feasible and cost-effective.

The population field needs increased commitment, appropriate policies, and adequate human and financial resources. If these conditions are met, population growth will slow, reproductive health will be improved, and the environment protected.

## 11. A new global green economy is needed

Better reproductive healthcare and decreased population pressures are essential but insufficient components of the transformed economy needed to preserve the environment. *The global community must cease the profligate and ecologically unsustainable exploitation of natural resources*. There is an urgent need for people everywhere, and especially high-consuming Americans, to advance a new economy that reduces consumption and the resulting waste and pollution, as well as preserves and restores natural systems (Hawken *et al*. 1999; Brown 2008).

It may be too late to avoid substantial climactic change, but we have much of the technology needed to minimize further, and even greater damage (Intergovernmental Panel on Climate Change 2007). We must move from a petroleum economy to an electric economy, powered mainly by wind, photovoltaic, geothermal and other renewable energy sources (Kammen 2006). In the meantime, we must dramatically decrease *per capita* energy use through energy-efficient appliances, lighting, buildings and homes. We must improve public transportation with high-speed electric trains, increase use of plug-in hybrid cars, and make our streets bicycle- and pedestrian-friendly (Brown 2008).

Livestock (e.g. beef, chicken, pork) production is the source of 18 per cent of greenhouse gases, second in importance only to the 21 per cent emitted by energy production and greater than the 14 per cent of emissions caused by all transportation activities. Lifestyle changes to minimize meat consumption would allow humans to consume the grains that are now fed to animals and diminish emission of greenhouse gasses (Fiala 2008).

We must replant forests to sequester carbon; curb deforestation for lumber, paper and fuel; and conserve and rebuild soil through appropriate plantings, limiting overgrazing and better farming practices. We can restore fisheries by limiting catches, establishing marine preserves, and protecting reefs and wetlands. Also, we must preserve and husband water resources through better irrigation practices, reduction of groundwater use, and increased use of composting toilets for the 2.6 billion people who now lack adequate sewage and sanitation facilities (Brown 2008; Worldwatch Institute 2009).

We must radically alter the world's current model of economic progress that is seemingly based upon ever-expanding consumption. Neither developed countries nor the developing world can afford this model. Developed countries must adopt a low-consumption economy, and developing countries must bypass the Western world's wasteful lifestyle and proceed directly to the same new economy.

While essential, achieving such change will be slow and expensive. Curbing and eventually halting population growth will buy the time needed to achieve systemic changes to the world's economy. Family planning and reproductive health programmes are affordable and feasible ways to help women avoid unintended pregnancies and slow population growth. Thus, increased access to family planning, combined with measures to increase the efficiency of consumption and protect the environment, offer a powerful strategy for helping ensure environmental sustainability (Speidel 2000).

As Lester Brown has noted:

The growth in resource consumption in China, now eclipsing that of the United States, provides convincing new reasons for shifting quickly from the fossil-fuel-based, automobile-centered, throwaway economy to a renewable energy-based, diversified-transport, reuse-recycle economy. In this restructuring, time is not on our side. It would be tempting to reset the clock, but we cannot. Nature is the timekeeper.

(L. R. Brown 2005, personal communication)

## Endnotes

1 The GBD is measured in disability-adjusted life years (DALYs), a measure that combines both years of life lost owing to premature death and years lived with disability. This approach to assessing the disease burden was developed by the World Bank and World Health Organization in the early 1990s. The DALY concept is far from perfect, but is widely used by economists and others, and provides one of the few methodologies for comparing diverse health conditions. In the reproductive health arena, this approach is seen as understating the impact of certain conditions, particularly those that are not disease-related.

2 It should be noted that improved standards of living will probably increase consumption and place additional stress on the environment. On the other hand, improvements in women's education, together with access to family planning information and services, will reduce population growth rates and pressures.

## References

[RSTB20090162C1] Abbasi-Shavazi M. J. 2002 Expert group meeting on completing the fertility transition. Population Division, Department of Economic and Social Affairs, United Nations Secretariat, New York

[RSTB20090162C2] Aghajanian A. Merhyar A. H. 1999 Fertility, contraceptive use and family planning program activity in the Islamic Republic of Iran. Int. Fam. Plann. Perspect. 25, 98–102 (doi:10.2307/2991948)

[RSTB20090162C3] Ahman E. Shah I. 2007 Unsafe abortion: global and regional estimates of the incidence of unsafe abortion and associated mortality in 2003 Geneva, Switzerland: World Health Organization

[RSTB20090162C4] Alan Guttmacher Institute 1999 Sharing responsibility: women, society and abortion worldwide New York, NY: Alan Guttmacher Institute

[RSTB20090162C5] Alan Guttmacher Institute 2000 Fulfilling the promise: public policy and U.S. family planning clinics New York, NY: Alan Guttmacher Institute

[RSTB20090162C6] Alan Guttmacher Institute 2005 State medicaid family planning eligibility expansions New York, NY: Alan Guttmacher Institute

[RSTB20090162C7] Ashford L. Clifton D. 2005 2005 women of our world Washington, DC: Population Reference Bureau See http://www.prb.org/pdf05/WomenOfOurWorld2005.pdf, accessed 3 January 2006

[RSTB20090162C8] Assadourian E. 2003 Economic growth inches up. In Vital Signs 2003 (eds Worldwatch Institute), pp. 44–45 New York, NY: W.W. Norton & Company

[RSTB20090162C9] Battisti D. S. Naylor R. L. 2009 Historical warnings of future food insecurity with unprecedented seasonal heat. Science 323, 240–244 (doi:10.1126/science.1164363) 19131626 10.1126/science.1164363

[RSTB20090162C10] Berer M. 2004 HIV/AIDS, sexual and reproductive health: intersections and implications for national programmes. Health Policy Plann. 19 (Suppl. 1), i62–i70 10.1093/heapol/czh046 15452016

[RSTB20090162C11] Bixby Center for Reproductive Health Research & Policy 2005 Final evaluation report of family PACT University of California, San Francisco, CA. See http://www.familypact.org/en/Research/reports.aspx, accessed 23 August 2006

[RSTB20090162C12] Black R. E. Allen L. H. Bhutta Z. A. de Onis M. Mathers C. Rivera J. 2008 Maternal and child undernutrition: global and regional exposures and health consequences. Lancet 371, 243–260 (doi:10.1016/S0140-6736(07)61690-0) 18207566 10.1016/S0140-6736(07)61690-0

[RSTB20090162C13] Blanc A. Curtis S. Croft T. 2001 Does contraceptive discontinuation matter? Meas. Eval. Bull. 1, 21–23

[RSTB20090162C14] Bongaarts J. 1994 Population policy options in the developing world. Science 263, 771–776 (doi:10.1126/science.8303293) 8303293 10.1126/science.8303293

[RSTB20090162C15] Bongaarts J. 2005 The causes of stalling fertility transitions. Population Council Policy Research Division Working Papers. New York, NY: Population Council

[RSTB20090162C16] Bongaarts J. 2009 Human population growth and the demographic transition. Phil. Trans. R. Soc. B 364, 2985–2990 (doi:10.1098/rstb.2009.0137) 19770150 10.1098/rstb.2009.0137 PMC2781829

[RSTB20090162C17] Bongaarts J. Mauldin W. P. Phillips J. F. 1990 The demographic impact of family planning programs. Stud. Fam. Plann. 21, 299–310 (doi:10.2307/1966918) 2075620

[RSTB20090162C18] Bongaarts J. O'Neill B. C. Gaffin S. R. 1997 Global warming policy: population left out in the cold. Environment 39, 40–41 10.1126/science.aat8680 30115798

[RSTB20090162C19] Braveman P. Egerter S. Marchi K. 1999 The prevalence of low income among childbearing women in California: implications for the private and public sectors. Am. J. Publ. Health 89, 868–874 (doi:10.2105/AJPH.89.6.868) 10.2105/ajph.89.6.868 PMC1508648 10358677

[RSTB20090162C20] Brindis C. Amaral G. Foster D. Biggs M. 2005 Cost–benefit analysis of the California family PACT program for calendar year 2002. See http://www.familypact.org/_resources/documents/Cost%20Benefit%20Final%20Report%20%201-28-05.pdf, accessed 5 March 2009

[RSTB20090162C21] Brown L. R. 2000 State of the world 2000: a Worldwatch Institute report on progress toward a sustainable society New York, NY: W.W. Norton & Company, Inc

[RSTB20090162C22] Brown L. R. 2004 Outgrowing the earth: the food security challenge in an age of falling water tables and rising temperatures New York, NY: W.W. Norton & Company, Inc

[RSTB20090162C23] Brown L. R. 2006 Plan B 2.0: rescuing a planet under stress and a civilization in trouble New York, NY: W.W. Norton & Company

[RSTB20090162C24] Brown L. R. 2008 Plan B 3.0: mobilizing to save civilization New York, NY: W.W. Norton & Company

[RSTB20090162C25] Camarota S. A. 2004 Economy slowed, but immigration didn't: the foreign-born population, 2000–2004 Washington, DC: Center for Immigration Studies

[RSTB20090162C26] Camarota S. A. 2005 Births to immigrants in America, 1970 to 2002 Washington, DC: Center for Immigration Studies

[RSTB20090162C27] Carr D. Khan M. 2004 The unfinished agenda: meeting the need for family planning in less developed countries Washington, DC: Population Reference Bureau

[RSTB20090162C28] Chabot M. Bradsberry M. Hulett D. Lewis C. 2006 Meeting the need for publicly funded contraceptive services, FY1999/00–FY2003/04. See http://www.familypact.org, accessed 23 August 2006

[RSTB20090162C29] Chao D. N. W. Allen K. B. 1984 A cost–benefit analysis of Thailand's family planning program. Int. Fam. Plann. Perspect. 10, 75–81 (doi:10.2307/2947609)

[RSTB20090162C30] Crane B. B. Hord Smith C. E. 2006 Access to safe abortion: an essential strategy for achieving the millennium development goals to improve maternal health, promote gender equality, and reduce poverty. Millennium Project, New York. See http://www.unmillenniumproject.org/documents/Crane_and_Hord-Smith-final.pdf, accessed 5 March 2009

[RSTB20090162C31] Cross H. Hardee K. Ross J. 2002 Completing the demographic transition in developing countries Washington, DC: POLICY Project

[RSTB20090162C32] Crossette B. 2004 Reproductive health and the millennium development goals: the missing link. Menlo Park. See http://www.hewlett.org/Programs/Population/Publications/crossettereport.htm, accessed 10 October 2005 10.1111/j.1728-4465.2005.00042.x 15828526

[RSTB20090162C33] Eastwood R. Lipton M. 2006 The role of fertility reduction in achieving the millennium development goals. Evidence presented to the U.K. All-Party Group on Population, Development and Reproductive Health. See http://www.appg-popdevrh.org.uk, accessed 7 June 2006

[RSTB20090162C34] Ehrlich P. 1968 The population bomb New York, NY: Ballantine

[RSTB20090162C35] Ehrlich P. Holdren J. 1971 The impact of population growth. Science 171, 1212–1217 (doi:10.1126/science.171.3977.1212) 5545198 10.1126/science.171.3977.1212

[RSTB20090162C36] Ehrlich P. R. Ehrlich A. H. 1990 The population explosion New York, NY: Simon and Schuster

[RSTB20090162C37] Ethelston S. Bechtel A. Chaya N. Kantner A. Vogel C. G. 2004 Progress and promises: trends in international assistance for reproductive health and population Washington, DC: Population Action International

[RSTB20090162C38] Fiala N. 2008 Meeting the demand: an estimation of future greenhouse gas emissions from meat production. Ecol. Econ. 67, 412–419 (doi:10.1016/j.ecolecon.2007.12.021)

[RSTB20090162C39] Finer L. B. Henshaw S. K. 2003 Abortion incidence and services in the United States in 2000. Perspect. Sex. Reprod. Health 35, 6–15 (doi:10.1363/3500603) 12602752 10.1363/3500603

[RSTB20090162C40] Finer L. B. Henshaw S. K. 2006 Disparities in rates of unintended pregnancy in the United States, 1994 and 2001. Perspect. Sex. Reprod. Health 38, 90–96 (doi:10.1363/3809006) 16772190 10.1363/psrh.38.090.06

[RSTB20090162C41] Finkle C. 2003 Ensuring contraceptive supply security. Outlook 20, 1–8

[RSTB20090162C42] Food and Agriculture Organization of the United Nations 2001 Global Forest Resources Assessment 2000 Rome: United Nations Economic and Social Council

[RSTB20090162C43] Food and Agriculture Organization of the United Nations 2008 The state of food insecurity in the world 2008 Rome: United Nations Economic and Social Council

[RSTB20090162C44] Food and Agriculture Organization of the United Nations 2009 The state of world fisheries and aquaculture 2008 Rome: United Nations Economic and Social Council

[RSTB20090162C45] Foster D. G. Biggs M. A. Amaral G. Brindis C. Navarro S. Bradsberry M. Stewart F. 2006 Estimates of pregnancies averted through California's family planning waiver program in 2002. Perspect. Sex. Reprod. Health 38, 126–131 (doi:10.1363/3812606) 16963385 10.1363/psrh.38.126.06

[RSTB20090162C46] Frost J. J. Sonfield A. Gold R. B. 2006 Estimating the impact of expanding medicaid eligibility for family planning services. Occasional Report No. 28. New York, NY: Guttmacher Institute See http://www.guttmacher.org/pubs/2006/08/16/or28.pdf, accessed 25 November 2008

[RSTB20090162C47] Frost J. J. Sonfield A. Gold R. B. Ahmed F. H. 2006 Estimating the impact of serving new clients by expanding funding for Title X New York, NY: Guttmacher Institute

[RSTB20090162C48] Germain A. Kyte R. 1995 The Cairo consensus: the right agenda for the right time New York, NY: International Women's Health Coalition

[RSTB20090162C49] Gillespie D. Maguire E. S. Neuse M. Sinding S. W. Speidel J. J. 2009 Making the case for U.S. International family planning assistance. See http://www.jhsph.edu/gatesinstitute/_pdf/policy_practice/Papers/MakingtheCase.pdf, accessed 21 February 2009 10.1016/S0140-6736(09)60837-0 19410699

[RSTB20090162C50] Green C. P. 1992 The environment and population growth: decade for action Baltimore, MD: Johns Hopkins University, Population Information Program

[RSTB20090162C51] Harte J. 2007 Human population as a dynamic factor in environmental degradation. Popul. Environ. 28, 223–236 (doi:10.1007/s11111-007-0048-3)

[RSTB20090162C52] Hawken P. Lovins A. B. Hunter Lovins L. 1999 Natural capitalism: creating the next industrial revolution Boston, MA: Little, Brown and Co

[RSTB20090162C53] Hirschman C. Tan J. E. Chamratrithirong A. Guest P. 1994 The path to below replacement-level fertility in Thailand. Int. Fam. Plann. Perspect. 20, 82–87 and 107 (doi:10.2307/2133509)

[RSTB20090162C54] Hollmann F. W. Mulder T. J. Kallan J. E. 2000 Methodology and assumptions for the population projections of the United States: 1999 to 2100. US Census Bureau See http://www.census.gov/population/www/documentation/twps0038.html, accessed 19 August 2005

[RSTB20090162C55] Hoodfar H. Assadpour S. 2000 The politics of population policy in the Islamic Republic of Iran. Stud. Fam. Plann. 31, 19–34 (doi:10.1111/j.1728-4465.2000.00019.x) 10765535 10.1111/j.1728-4465.2000.00019.x

[RSTB20090162C56] Intergovernmental Panel on Climate Change 2007 Climate change 2007: Synthesis report. Summary for policymakers. See http://www.ipcc.ch/pdf/assessment-report/ar4/syr/ar4_syr_spm.pdf, accessed 16 February 2009

[RSTB20090162C57] Jones R. K. Purcell A. Singh S. Finer L. B. 2005 Adolescents' reports of parental knowledge of adolescents' use of sexual health services and their reactions to mandated parental notification for prescription contraception. J. Am. Med. Assoc. 293, 340–348 (doi:10.1001/jama.293.3.340) 10.1001/jama.293.3.340 15657327

[RSTB20090162C58] Kammen D. M. 2006 The rise of renewable energy. Scient. Am. 295, 84–93 10.1038/scientificamerican0906-84 16925040

[RSTB20090162C59] Kendall H. 1992 World scientists' warning to humanity. Union of Concerned Scientists, Cambridge, MA See http://www.ucsusa.org/ucs/about/page.cfm?pageID=1009, accessed 6 December 2005

[RSTB20090162C60] Kent M. M. Mather M. 2002 What drives U.S. population growth? Washington, DC: Population Reference Bureau

[RSTB20090162C61] Kingstone S. 2005 Amazon destruction accelerating. BBC News, 19 May 2005. See http://news.bbc.co.uk/2/hi/americas/4561189.stm

[RSTB20090162C62] Larsen J. 2001 Iran's birth rate plummeting at record pace: success provides a model for other developing countries. Earth Policy Institute See http://earth-policy.org/Updates/Update4ss.htm, accessed 5 January 2005

[RSTB20090162C63] Levine R. Langer A. Birdsall N. Matheny G. Wright M. Bayer A. 2006 Contraception. In Disease control priorities in developing countries (ed. Jamison D. T.), pp. 1075–1090 Washington, DC: World Bank

[RSTB20090162C64] Markham V. D. Steinzor N. 2006 U.S. National Report on population and the environment. New Canaan, CT: Center for Environment and Population

[RSTB20090162C65] McDevitt T. M. 1999 World population profile: 1998. US Census Bureau, Washington, DC See http://www.census.gov/ipc/prod/wp98/wp98.pdf, accessed 7 October 2005

[RSTB20090162C66] McFalls J. A. Jr 2007 Population: a lively introduction. Popul. Bull. 62, 1–31

[RSTB20090162C67] McGranahan G. Balk D. Anderson B. 2007 The rising tide: assessing the risks of climate change and human settlements in low elevation coastal zones. Environ. Urban. 18, 17–37

[RSTB20090162C68] McIntosh C. A. Finkle J. L. 1995 The Cairo conference on population and development: a new paradigm? Popul. Dev. Rev. 21, 223–260 (doi:10.2307/2137493)

[RSTB20090162C69] Millennium Ecosystem Assessment 2005 Ecosystems and human well-being: synthesis Washington, DC: Island Press

[RSTB20090162C70] Mosher W. D. Chandra A. Jones J. 2005 Sexual behavior and selected health measures: men and women 15–44 years of age, United States, 2002. Adv. Data Vital Health Stat. 362, 1–56 16250464

[RSTB20090162C71] National Academy of Sciences, National Academy of Engineering and Institute of Medicine 1993 Population summit of the World's scientific academies Washington, DC: The National Academies Press

[RSTB20090162C72] National Family Planning and Reproductive Health Association 2009 The final countdown: the last year of the Bush Administration Federal Legislative and Regulatory Action on Reproductive Health in 2008 Washington, DC: National Family Planning and Reproductive Health Association

[RSTB20090162C73] Organisation for Economic Co-operation and Development Assistance Committee 2008 Aid targets slipping out of reach? Paris, Organisation for Economic Co-operation and Development See http://www.oecd.org/dataoecd/47/25/41724314.pdf, accessed 16 February 2009

[RSTB20090162C74] Palermo M. P. 2005 Brazil losing fight to save the Amazon. Reuters, 23 May 2005. See http://www.enn.com/top_stories/article/1609

[RSTB20090162C75] Passel J. S. 2006 The size and characteristics of the unauthorized migrant population in the U.S.: estimates based on the March 2005 current population survey Washington, DC: Pew Hispanic Center

[RSTB20090162C76] Perry M. J. Mackun P. J. 2001 Population change and distribution: 1990 to 2000. U.S. Census Bureau, Washington, DC See http://www.census.gov/prod/2001pubs/c2kbr01-2.pdf, accessed 9 December 2005

[RSTB20090162C77] Population Division of the Department of Economic and Social Affairs of the United Nations Secretariat 2005 World population prospects: the 2004 revision and world urbanization prospects: the 2003 revision. See http://esa.un.org/unpp/, accessed 20 June 2005

[RSTB20090162C78] Population Division of the Department of Economic and Social Affairs of the United Nations Secretariat 2007 World population prospects: the 2006 revision. See http://www.un.org/esa/population/publications/wpp2006/wpp2006.htm, accessed 20 March 2007

[RSTB20090162C79] Population Division of the Department of Economic and Social Affairs of the United Nations Secretariat 2009 World population prospects: the 2008 revision. See http://www.un.org/esa/population/unpop.htm, accessed 12 March 2009

[RSTB20090162C80] Population Reference Bureau 2008a 2008 World Population Data Sheet. Washington, DC. See http://www.prb.org/pdf08/08WPDS_Eng.pdf, accessed 14 December 2008

[RSTB20090162C81] Population Reference Bureau 2008b Family planning worldwide: 2008 data sheet. See http://www.prb.org/pdf08/fpds08.pdf, accessed 14 December 2008

[RSTB20090162C82] Potts M. 1997 Sex and the birth rate. Popul. Dev. Rev. 23, 1–39 (doi:10.2307/2137459)

[RSTB20090162C83] Rosenfield A. G. Hemachudha C. Asavasena W. Varakamin S. 1971 Thailand: family planning activities 1968 to 1970. Stud. Fam. Plann. 2, 181–192 (doi:10.2307/1964903) 5128532

[RSTB20090162C84] Ross J. Stover J. 2001 The family planning program effort index: 1999 cycle. Int. Fam. Plann. Perspect. 27, 119–129 (doi:10.2307/2673833)

[RSTB20090162C85] Sedgh G. Hussain R. Bankole A. Singh S. 2007 Women with an unmet need for contraception in developing countries and their reasons for not using a method New York, NY: Guttmacher Institute

[RSTB20090162C86] Singh S. Darroch J. E. Vlassoff M. Nadeau J. 2003 Adding it up: the benefits of investing in sexual and reproductive health care New York, NY: Guttmacher Institute

[RSTB20090162C87] Sonfield A. 2003 Preventing unintended pregnancy: the need and the means New York, NY: Guttmacher Institute

[RSTB20090162C88] Sonfield A. Gold R. B. 2005 Public funding for contraceptive, sterilization and abortion services, FY1980–2001 New York, NY: Guttmacher Institute

[RSTB20090162C89] Speidel J. J. 2000 Environment and health. 1. Population, consumption and human health. Can. Med. Assoc. J. 163, 551–556 11006767 PMC80465

[RSTB20090162C90] Speidel J. J. 2005 Population donor landscape analysis for review of Packard Foundation International Grantmaking in Population, Sexual and Reproductive Health and Rights. The David and Lucile Packard Foundation See http://www.packard.org/assets/files/population/program%20review/pop_rev_speidel_030606.pdf, accessed 13 July 2006

[RSTB20090162C92] Trussell J. 2004 The essentials of contraception: efficacy, safety, and personal considerations. In Contraceptive technology (eds Hatcher R. A. Trussell J. Stewart F. Nelson A. L. Cates W. Guest F. Kowal D.), pp. 21–252 New York, NY: Ardent Media

[RSTB20090162C93] US Census Bureau 2006 Statistical abstract of the United States: 2007. Washington, DC. See http://www.census.gov/prod/www/statistical-abstract.html, accessed 12 February 2007

[RSTB20090162C94] US Census Bureau and Population Division 2006 Annual estimates of the population for the United States, Regions, and States and for Puerto Rico: April 1, 2000 to July 1, 2006 (NST-EST2006-01). Washington, DC. See http://www.census.gov/popest/states/NST-ann-est.html, accessed 15 January 2007

[RSTB20090162C95] UN Millennium Project 2005 Investing in development: a practical plan to achieve the millennium development goals New York, NY: United Nations Development Programme

[RSTB20090162C96] UNAIDS 2005 Questions and answers: international programmes, initiatives and funding issues. See http://www.unaids.org/html/pub/una-docs/q-a_i_en_pdf.pdf, accessed 18 October 2005

[RSTB20090162C97] UNAIDS 2007 Financial resources required to achieve universal access to HIV prevention, treatment, care and support Geneva, Switzerland: UNAIDS

[RSTB20090162C98] UNFPA 1995 Summary of the ICPD programme of action. See http://www.unfpa.org/icpd/summary.cfm, accessed 10 October 2005

[RSTB20090162C99] United Nations Department of Economic and Social Affairs Population Division 1999 The world at six billion New York, NY: United Nations

[RSTB20090162C100] United Nations Department of Economic and Social Affairs Population Division 2004 World population to 2300 New York, NY: United Nations

[RSTB20090162C101] United Nations Department of Economic and Social Affairs Population Division 2007 World contraceptive use 2007. New York. See http://www.un.org/esa/population/publications/contraceptive2007/contraceptive_2007_table.pdf, accessed 14 December 2008

[RSTB20090162C102] United Nations Development Programme, United Nations Environment Programme, World Bank and World Resources Institute 2002 A guide to world resources 2002–2004: decisions for balance, voice, and power Washington, DC: World Resources Institute

[RSTB20090162C103] United Nations Economic and Social Council 2005 Flow of financial resources for assisting in the implementation of the programme of action of the International Conference on Population and Development. Report of the Secretary-General to the 37th Session of the Commission on Population and Development, 22–26 March 2004, United Nations, New York.

[RSTB20090162C104] United Nations Economic and Social Council 2009 The flow of financial resources for assisting in the implementation of the programme of action of the International Conference on Population and Development. Report of the Secretary-General to the 42nd Session of the Commission on Population and Development, 30 March–3 April 2009, United Nations, New York.

[RSTB20090162C105] United Nations Environment Programme 2006 Challenges to international waters: regional assessments in a global perspective Nairobi, Kenya: United Nations Environment Programme

[RSTB20090162C106] Vlassoff M. Singh S. Darroch J. E. Carbone E. Bernstein S. 2004 Assessing costs and benefits of sexual and reproductive health interventions New York, NY: Guttmacher Institute

[RSTB20090162C107] Westoff C. F. 2001 Unmet need at the end of the century Calverton: O.R.C. Macro

[RSTB20090162C108] Westoff C. F. Bankole A. 2000 Trends in the demand for family limitation in developing countries. Int. Fam. Plann. Perspect. 26, 56–62 and 97 (doi:10.2307/2648268)

[RSTB20090162C109] Wilson E. O. 2002 The future of life New York, NY: Alfred A. Knopf

[RSTB20090162C110] Wilson E. W. Kehoe K. 2000 Environmental organizations and international population assistance. Unpublished report submitted to the Summit, Packard & Hewlett Foundations

[RSTB20090162C111] World Bank 2005 World development indicators. See http://www.worldbank.org/data/wdi2005/wditext/home.htm, accessed 11 October 2005

[RSTB20090162C112] World Resources Institute 1998 World Resources 1998–99. A guide to the global environment: environmental change and human health New York, NY: Oxford University Press

[RSTB20090162C113] Worldwatch Institute 1999 Our demographically divided world: rising mortality joins falling fertility to slow population growth Washington, DC: Worldwatch Institute See http://www.worldwatch.org/press/news/1999/04/08/, accessed 20 June 2005

[RSTB20090162C114] Worldwatch Institute 2009 State of the world 2009: into a warming world New York, NY: W.W. Norton & Co

